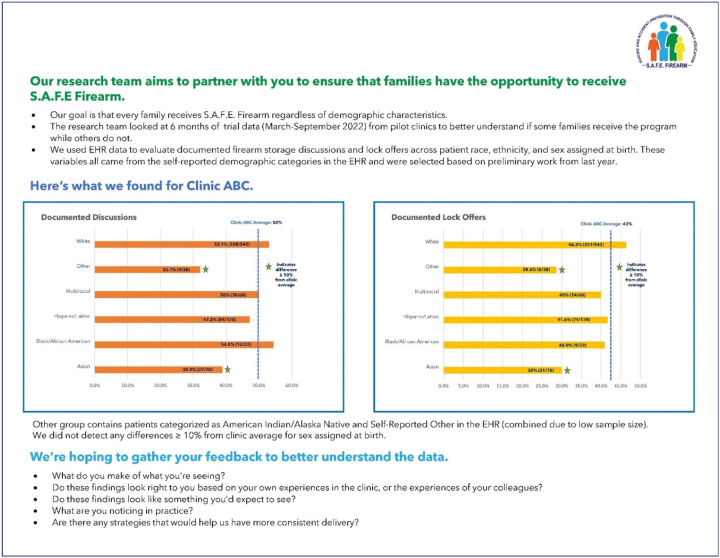# Opportunities to Advance Equity Through Implementation Strategy Design

**DOI:** 10.21203/rs.3.rs-4773990/v1

**Published:** 2024-09-02

**Authors:** KATELIN HOSKINS, Melissa Maye, Leslie Wright, Shari Jager-Hyman, Courtney Benjamin Wolk, Brian Ahmedani, Jennifer M Boggs, Christina Johnson, Kristin Linn, LeeAnn Quintana, Celeste Pappas, Rinad S Beidas

**Affiliations:** University of Pennsylvania School of Nursing; Henry Ford Health System; Kaiser Permanente Colorado Institute for Health Research; University of Pennsylvania Perelman School of Medicine; University of Pennsylvania Perelman School of Medicine; Henry Ford Health System; Kaiser Permanente Colorado Institute for Health Research; Northwestern University Feinberg School of Medicine; University of Pennsylvania Perelman School of Medicine; Kaiser Permanente Colorado Institute for Health Research; Henry Ford Health System; Northwestern University Feinberg School of Medicine

**Keywords:** health equity, hybrid effectiveness-implementation trial, implementation strategy, facilitation, audit and feedback

## Abstract

**Background:**

Implementation strategies are potential tools for advancing equity goals in healthcare. Implementation scientists have increased attention to the integration of equity considerations into implementation research, but limited concrete guidance is available for developing implementation strategies to improve equity.

**Main:**

In parallel to an active hybrid effectiveness-implementation trial in two large health systems, our research team explored potential inequities in implementation across four non-study clinics, developed equity focused audit and feedback procedures, examined the feasibility of our approach, and identified design insights that could be tested in future work to inform equitable program scale-up. Based on our experiences deploying these strategies in pilot format, our research team identified key complexities meriting further examination in future work. These considerations are vital given the dearth of guidance on delivering feedback to clinicians in efforts to improve equity. Key takeaways include the importance of understanding local data culture, engaging constituents in co-design for the full feedback cycle, leveraging feedback for shared discourse, and centering multi-level strategies as part of robust implementation approaches.

**Conclusion:**

Prioritizing health equity in implementation science requires that research teams probe, interrogate, and innovate – and in doing so, grapple with central conceptual and pragmatic considerations that arise in the design of implementation strategies. Our work emphasizes the value of bidirectional and continuous learning.

## BACKGROUND

Implementation strategies, or the methods used to enhance the adoption, sustainability, and scale-up of evidence-based interventions (EBIs),^1^ are potential tools for advancing equity goals in healthcare. Implementation scientists have increased attention to the integration of equity considerations into all features of implementation research,^2^ but limited concrete guidance is available for developing or adapting implementation strategies to improve equity. Thus, we share our research team’s experience designing an equity focused implementation strategy to inform future program scale-up.

Adolescent and child Suicide Prevention in Routine clinical Encounters (ASPIRE) is a hybrid effectiveness-implementation type III trial that tested two approaches to implement an evidence-based rearm storage program as a universal suicide prevention strategy in 30 pediatric primary care clinics across two racially, ethnically, and geographically diverse health systems.^3^ The program, *S.A.F.E. Firearm*, includes a brief discussion with parents or guardians on secure firearm storage and distribution of free cable locks during all well-child visits for children ages 5–17. An electronic health record (EHR) nudge (i.e., prompt) incorporated into pediatric clinicians’ well-child visit templates (Nudge) and the EHR nudge combined with practice facilitation (Nudge+) were the two implementation strategies tested to promote clinician delivery of *S.A.F.E. Firearm*.

Our team engaged in a two-phase pilot project to enhance attention to health equity within our implementation trial and explore how equity considerations could be addressed prospectively to support consistent deployment of *S.A.F.E. Firearm* in future implementation and scale-up efforts. In the first phase, we conducted a multi-method implementation pilot study^4^ across five pediatric primary care clinics from the participating health systems. We examined equitable implementation of *S.A.F.E. Firearm* across key patient characteristics identified as salient by the literature and our clinical partners: medical complexity, sex, race, and ethnicity.^4^

In the second phase of our project, we honed in on implementation strategy design. Our team aimed to understand how elements of facilitation, as one of the implementation approaches being tested in the trial, might be adapted to promote equity. Facilitation builds organizational capacity for improvement and troubleshoots barriers to implementation.^5^ The approach includes multi-faceted implementation strategies, including partner engagement and audit and feedback (i.e., A&F; collecting clinician performance data and sharing it with the clinician, typically with comparison to targets).^5^ Our team identified A&F as the most practical implementation strategy within facilitation to adapt. Within this second phase, we continued to quantitatively explore potential inequities in implementation, developed equity focused A&F procedures within facilitation, examined the feasibility of our approach, and identified design insights that could be tested in future work. Here we describe our process, key reflections, and opportunities for other research teams to expand upon our efforts to promote equity within implementation strategy design.

## MAIN TEXT

### Process

Step 1: Literature Review and Expert Consultation. We completed a PubMed literature review in summer 2022 to identify articles specific to implementation, facilitation, A&F, and disparities. To identify gray literature, we conducted a Google search and reviewed references from the U.S. Department of Veterans Affairs (VA) Quality Enhancement Research Initiative (QUERI) Facilitation Guide.^5^ Findings were sparse, and we did not identify clear guidance that specified how to operationalize facilitation to support equitable implementation. Moreover, while A&F is commonly used to improve care quality, little is known about optimal approaches to deliver feedback on inequities to clinicians, nor the actual effectiveness of A&F in improving equity. To expand on this search, we also consulted with one leading facilitation and health equity expert and the VA Quality Enhancement Research Initiative (QUERI) Facilitation Workgroup.

Step 2: Auditing Clinic-Level Data: We examined six months of data (March-September 2022) from patients ages 5–17 seen in four pilot clinics. Outcomes were clinician-documented delivery (yes/no) of each component of *S.A.F.E. Firearm* during well child visits: secure storage discussion and lock offer. Descriptive statistics were used to analyze the percentage of documented “yes” for each outcome stratified across race and ethnicity and sex assigned at birth in aggregate at the clinic-level. Groups with small numbers (n < 5) were combined with the Other race group. We set a 10 percentage point clinical significance threshold to mirror the main trial^3^ and considered differences in program delivery ≥ 10 percentage points from the clinic average to be clinically meaningful. Discussions with health system leaders and previous studies^6^ informed the selection of the trial’s 10 percentage point clinical significance margin.

Step 3: Framing Out Feedback: Aligned with the overall spirit of facilitation, we explicitly took a curious, collaborative, and nonjudgmental stance to developing equity focused A&F. We drew from the Health Equity Implementation Framework^7^ to situate our approach, identifying multi-level factors that could impact *S.A.F.E. Firearm* delivery within the clinical encounter. For example, innovation factors like limited translation of *S.A.F.E. Firearm* program materials to languages other than Spanish could impact implementation. With these and other health equity domains^7^ in mind, we were eager to feed back the audit data from Step 2.

Step 4: Operationalizing Feedback Procedures: The facilitation team (C.B.W., S.J.H., M.M., L.W.) internally iterated procedures for equity focused A&F. First, we identified local clinic champions within the pilot clinics (all physicians, N = 4) to receive feedback. These champions within the pilot clinics were especially open to exploring new implementation approaches, and we viewed feedback sessions as a potential opportunity for rich discourse. We hoped to triangulate their insights with the six months of pilot clinic audit data. We drafted emails with invitations to meet one-on-one with the trained facilitator – who was a member of the research team and employed by each respective health system but did not provide clinical care within the individual clinics (M.M., L.W.) – to discuss results. Second, we developed a data visualization tool with graphical and text summaries (see Fig. 1) to communicate our results.^8^ In doing so, we focused on minimizing cognitive load by reducing jargon and clearly articulating our intentions (i.e., sharing data to ensure we are not unintentionally creating inequities between patient subpopulations). Third, we developed specific questions for further inquiry: “What do you make of what you’re seeing?”, “Do these findings look right to you based on your own experiences in the clinic and the experiences of your colleagues?”, and “What are you noticing in practice?” We also integrated an acknowledgment that differences might not be due to clinician or organizational behavior but may relate to how the *S.A.F.E. Firearm* program was developed (for example, we did not adapt materials to directly address parents who own firearms for protection in the context of community violence).

Step 5: Completing feedback sessions: Lastly, we completed structured feedback sessions and engaged in discussions directly following feedback over the course of a 30-minute virtual meeting. We aimed to present data during the feedback in an unbiased manner with transparency on data collection and analytic methods. Three clinic champions participated.

### Reflections and Design Recommendations

Based on our experiences providing feedback to clinic champions across both health systems, our research team identified key complexities that merit further examination in future work. These considerations are vital given the dearth of guidance on delivering feedback to clinicians in efforts to improve equity.

#### Pragmatic Considerations:

Understanding Local Data Culture to Inform User-Centered Design. Our process revealed pragmatic challenges with the clinic-level data audit. Given the preliminary nature of the work, the well-child visit numbers were small when stratified across race and ethnicity, and the overall distribution of race and ethnicity varied substantially across clinics. Clinic champions practicing in clinics with less diverse populations had difficulty interpreting the strength of what our study team deemed clinically significant differences due to the small sample sizes of the subpopulations. Relatedly, some clinic champions were interested in reports that included analyses examining statistically significant differences between various subpopulations, in contrast to our descriptive analyses. While multi-level modeling was used in our pilot study, we found that these analyses were time intensive, and thus, not feasible for replication in a prospective and real-time A&F format; reporting of results required highly technical nuance that was impractical to describe during these brief meetings with clinicians; and interpretations in these full sets of analyses would still be limited by race and ethnicity distributions.^4^ Other considerations included alternative levels of analyses, specifically at the level of individual clinicians instead of clinics, but we anticipated logistical challenges related to meeting with every clinician. Clinicians also suggested consideration of other variables that that may be associated with program delivery, like medical complexity and language, in future iterations of equity focused A&F.

Notably, however, a focus on justifying data outputs is not unique to our A&F use case, and perceptions of data credibility may shape whether clinicians reflect on the results and/or deflect practice change.^8–11^ While data signals must be interpreted cautiously, Desveraux and colleagues underscore that “imperfect data can still provide insight.”^9 (p325)^ Understanding local data culture – that is, the collective beliefs and behaviors related to data use within an organization^12^ – may guide A&F use, particularly in the context of clinicians’ exposures to quality improvement data for disparity reduction (with an eye toward potential oversaturation with data). Capitalizing on clinicians’ insights into the data culture of their clinics has potential to shape data audit inputs and subsequent feedback in efforts to strengthen the credibility and potential impact of A&F.

Enhanced attention to data culture and context points to the value of applying human-centered design principles and methods to implementation strategy design.^13^ Operationalizing implementation strategies requires attention to *usability* from the perspective of end users (i.e., clinicians) to optimize fit, effectiveness, and sustainability.^13,14^ Moreover, new theoretical development for A&F suggests that ownership and buy-in lead to trust and capability, emphasizing the value of collaboration in audit design.^15^ Co-designing an equity focused A&F strategy would elicit clinicians’ preferred approaches for each stage of the A&F cycle, like clinical significance thresholds in the goal setting stage, equity relevant variables (e.g., language) in the data collection stage, and level of analyses (e.g., individual- or clinic-level messaging) in the feedback stage. Iterative prototyping of the A&F report and delivery format (e.g., individual or group face-to-face discussions, emailed reports, equity dashboards) also has potential to enhance quality and engagement, which is important given that information design and delivery has been hypothesized to impact A&F effectiveness.^11,16^ Designing with an eye toward sustainability – what can be maintained by the organization after the trial ends – is also imperative.

#### Conceptual Considerations:

Recognizing Clinician Capacity to Address Potential Multilevel Drivers. Within our dataset, we suspected that varied factors across multiple levels (e.g., societal, organizational, clinician, and innovation)^7^ could be potential determinants of inequitable implementation. With that framing, conceptual constraints bounded this initial pilot project of A&F as a discrete equity promoting change strategy. First, our feedback sparked reflection and highlighted potential barriers to implementation but did not confirm multi-level drivers of outcome differences. Clinic champions were curious about the impact of routinization effects for clinicians with older patient panels, use of languages other than English, and competing health care needs as potential factors. These insights opened potential lines of additional inquiry for our team rather than cleanly answering why differences occurred over the audit period. As such, our team was limited in offering immediate targeted actions and follow-up plans to clinic champions to improve program underuse within the context of facilitation. Second, because A&F primarily targets individual-level domains (i.e., knowledge, intention, skills), A&F functions as a clinician- or team-level implementation strategy. The strategy provides an important opportunity for clinicians to cue action and change what is within their control (e.g., biased decision-making) but will not modify systemic barriers, like visit times and volume pressures or quick access to interpreter services.

When designing equity focused A&F, the strategy may most optimally be leveraged as a reflective tool^9^ that facilitates bidirectional discourse between researchers, clinicians, and even organizational leaders with influence to enact change. A&F may then serve a knowledge-generating function, in addition to enhancing nimbleness and responsiveness to existing challenges and changing context. Additional time in our feedback sessions with other clinic constituents (including parents) may have allowed for more robust and collaborative efforts to generate explanations and corresponding actions for findings related to organizational-, clinician-, innovation-level or other factors affecting implementation. For example, if we elicited that clinicians’ assumptions about the program’s cultural fit impacts communication and subsequent program delivery,^17^ clinician training or facilitation coaching might be adapted to explicitly incorporate principles of cultural responsiveness. However, if discussion of A&F results points to systemic or organizational barriers, new learnings might serve as a springboard for strategy development targeted at determinants beyond the individual-level (e.g., insufficient visit time to address the acute and surging mental health needs of teen girls).

Considering equity focused A&F as part of a suite of implementation approaches may more comprehensively address challenges that cannot readily be modified by clinicians. Multi-level barriers may indeed exceed clinician capacity for change, even for those clinicians who endorse the credibility and value of the data or interpretations presented in reports. As such, targeting organizational leaders with clinic-level audit data may better support the system-level changes needed for clinicians to optimize their professional practice and advance equitable outcomes. Moreover, shifting the onus to researchers, organizational leaders, and quality improvement teams has potentially important repercussions for clinician morale in times of high burnout, especially given that clinicians often serve as “shock absorbers,” absorbing “interconnected structural demands” with costs to their own wellbeing.^18 (p. 1)^ Design of multi-level implementation strategies targeting societal- and organizational-level determinants and rapid adaptations to changing context are critical future directions for implementation research.^19^

## CONCLUSION

Prioritizing health equity in implementation science requires that research teams probe, interrogate, and innovate – and in doing so, grapple with central pragmatic and conceptual challenges. Whether A&F can improve equity requires further investigation, but our research team’s experience generated preliminary insights on the use of A&F within practice facilitation, informing opportunities for equitable program scale-up. Key takeaways include the importance of understanding local data culture, engaging constituents in co-design for the full feedback cycle, leveraging feedback for shared discourse, and centering multi-level strategies as part of robust implementation approaches. Our work amplifies the value of continuous, bidirectional learning in designing implementation strategies to advance equity goals.

## Figures and Tables

**Figure 1:**